# From the Mediterranean to the Sea of Japan: The Transcontinental Odyssey of Autoinflammatory Diseases

**DOI:** 10.1155/2013/485103

**Published:** 2013-07-23

**Authors:** Donato Rigante, Bruno Frediani, Mauro Galeazzi, Luca Cantarini

**Affiliations:** ^1^Institute of Pediatrics, Università Cattolica Sacro Cuore, Largo A. Gemelli 8, 00168 Rome, Italy; ^2^Research Center of Systemic Autoimmune and Autoinflammatory Diseases, Rheumatology Unit, Policlinico Le Scotte, University of Siena, Viale Bracci 1, 53100 Siena, Italy

## Abstract

Autoinflammatory diseases are comprehensively caused by aberrant production of proinflammatory cytokines and are revealed by cyclically and spontaneously occurring inflammatory events. Over the last decade, there has been a revolution in the understanding of periodic fever syndromes, cryopyrinopathies, and skin disorders with pyogenic, granulomatous, or dystrophic features, which have been recognized across different countries spanning from the Mediterranean basin to the Japanese archipelago. Many children and adults with autoinflammatory diseases continue to elude diagnosis, and the diagnostic delay of many years puts these patients at risk of long-term severe complications, such as amyloidosis. Any hint of suspicion of autoinflammatory disease thus needs to be highlighted in various medical specialties, and this review examines their frequencies around the world, trying to match them with geographic location, ethnic and genetic data, in an attempt to realize a geoepidemiologic map for most of these conditions.

## 1. Introduction

Inflammation is traditionally classified among the nonspecific defense mechanisms of the human body: this is partly true for acute inflammation, which can be fatal in vital districts such as the brain and heart and completely untrue for chronic inflammation, which represents the largest sector of human pathology and is a central component of the specific immune response, involving several cell types and hundreds of different molecules with innumerable interactions among one another. Autoinflammatory diseases (AIDs) were so named in 1999 to characterize a group of hereditable monogenic conditions, defined by recurrent episodes of systemic and organ-specific sterile inflammation caused by mutations in proteins involved in the innate immune response [1]: these include cytokine receptors, receptor antagonists, and components of the inflammasome, that is, a set of intracellular protein complexes that enable the autocatalytic activation of inflammatory caspases, driving the release of interleukin-1 (IL-1)/IL-1 family members into the blood stream [2]. By specifically blocking IL-1, we have learned a great deal about the role of this cytokine in inflammation and the central role of caspase activation in the pathogenesis of various AIDs, which have been recognized across different countries spanning from the Mediterranean sea to East Asia [3].

## 2. Familial Mediterranean Fever

Familial Mediterranean fever (FMF) is the most common AID, predominating in people living around the Eastern Mediterranean basin [4] and is caused by loss-of-function mutations within the *MEFV* gene: pyrin is the product of *MEFV* and is an intracellular regulator of IL-1 production [5]. In Sephardic Jews, Turks, Arabs, and Armenians, the carrier rate for a mutant *MEFV* allele ranges from 1 : 3 (Armenia) to 1 : 6, the highest rates reported for an autosomal recessive disorder [6]. Despite the high frequency of these recessive alleles, the prevalence of patients with a definite diagnosis of FMF is much lower than expected, and the spectrum of clinical symptoms may differ considerably from one patient to another, depending on specific mutations, with a variegated risk of secondary renal amyloidosis [7]. This surprisingly increased normal carrier rate cannot be explained by intense inbreeding or elevated genetic drift alone, and the presence of recurrent mutations in *MEFV *suggests a common ancestral origin, implying a strong selection pressure. FMF is classically described as autosomal recessive, but many patients have only one mutated allele justifying a dominant pattern of inheritance, which might confer an evolutionary advantage in resisting an endemic pathogen [8]. *MEFV* mutations probably arose in pre-Biblical times and were disseminated into various populations, where they are still found today, with nonuniform distribution: Jews, being genetically isolated, might represent the most likely candidate population for the greatest number of founder effects in *MEFV *[9]. Jalkh et al. estimated the ages of the most recent common ancestor for the *MEFV* mutations M694V, M694I, V726A, M680I, and E148Q to be 7000, 8500, 15000, 23000, and 30000 years B.C., respectively [10]. However, FMF can be seen in other ethnic groups as well, as in the Japanese population, albeit at a much lower frequency [11]. The hypothesis that other genetic systems affect the expression of FMF is supported by evidence that clinical symptoms of FMF-affected migrants living in Germany resemble those observed in their home country [12]. In addition, country of origin is the key risk factor for amyloidosis in FMF, and a patient's country should be considered in addition to *MEFV *genotype as an indication for prophylactic colchicine, an alkaloid extracted from lily plants, which is the most efficacious drug in FMF [13].

Table 1 lists the general details of FMF. Patients with the disease suffer lifelong self-limited recurrent bouts of fever and pain in the serosal and synovial membranes [14], with a periodicity varying from weekly to quarterly [15]. Diagnosis of FMF remains clinical and requires information about ethnic background, family history, and response to colchicine, since specific laboratory tests are not available [16]; genetic diagnosis can be confirmed by the presence of two mutations in the *MEFV* gene in at least 70% of patients with overt FMF, but heterozygous mutation carriers can also suffer from incomplete or even typical disease [17]. Colchicine's inhibitory effect on IL-1 release and its prophylactic role in preventing the recurrence of FMF attacks were discovered serendipitously [18]; however, alternative medications such as IL-1 blockers have been shown to be highly effective in poor responders to colchicine [19].

## 3. Mevalonate Kinase Deficiency 

Mevalonate kinase deficiency (MKD) is due to mutations in the *MVK* gene, encoding the second enzyme of mevalonate pathway, which results in abnormal enzymatic activity and subsequent shortage of downstream compounds, as serum cholesterol [20]. The disease was initially recognized in the Netherlands, then in people from different countries of northwestern Europe, in whom there is a carriage rate of 1 : 350 for the V377I* MVK *mutation [21]. Vuch et al. have hypothesized a selective advantage for heterozygote carriers of the most frequent *MVK* mutations in those countries where the diet is characterized by high consumption of saturated animal fats, rich in cholesterol [22]. MKD usually starts in childhood with abrupt febrile flares, skin or joint symptoms, and severe gastrointestinal complaints, sometimes induced by vaccinations or viral infections [23]. Highly characteristic of MKD are systemic antibiotic-resistant inflammation, elevated serum IgD in any phase of the disease, increased circulating IgM^−^IgD^+^ B lymphocytes, and increased urinary excretion of mevalonic acid during febrile flares [24]. General details of MKD are described in Table 2. Diagnosis of MKD is clinical, though genetic confirmation is required, mostly when serum IgD level is within the normal range. Treatment may require nonsteroidal anti-inflammatory drugs or corticosteroids during attacks, but great attention is now being given to IL-1 antagonists [25, 26], due also to the increasing evidence that IL-1 overproduction is fundamentally involved in the pathophysiology of this condition [27].

## 4. Tumor Necrosis Factor Receptor-Associated Periodic Syndrome

Tumor necrosis factor receptor-associated periodic syndrome (TRAPS) was originally described in a family of Irish and Scottish pedigree and initially thought to be a tumor necrosis factor (TNF) receptor-mediated disease, resulting from the failure of the TNF receptor to insert itself into cell membrane [28]. This is the most common dominant form of AID in Europe, historically known as “familial Hibernian fever,” from the ancient Latin name “Hibernia” given to Ireland: different mechanisms, from TNF receptor misfolding to abnormal trafficking, have been reported to explain TRAPS at a pathogenic level, leading to mutated TNF receptor accumulation inside cells and causing increased activation of reactive oxygen species and subsequent IL-1 release [29]. In TRAPS patients, the interpretation of *TNFRSF1A* mutations might be challenging: for instance, P46L substitution occurs in up to 20% of clinically asymptomatic West African individuals, which suggests that it represents a polymorphism rather than a disease-causing mutation, and R92Q substitution, relatively common in the Caucasian population, is a low-penetrance variant, which could have a weak contribution to disease expression. Moreover, *TNFRSF1A* mutations affecting TNF receptor shedding from cell membranes might potentially generate a selective advantage related to an increased antibacterial capacity [30].

The clinical picture is protean, characterized by long-lasting febrile episodes, differently from other AIDs and troublesome for both patients and clinicians [31]. General details are listed in Table 3. Genotype analysis is required for diagnosis of TRAPS, while prognosis is mainly determined by the risk of renal amyloidosis, which can be observed in 25% of patients with peculiar *TNFRSF1A* mutations [32]. Treatment with corticosteroids alleviates the inflammatory symptoms of TRAPS, but does not affect the frequency of attacks. The antitumor necrosis factor inhibitor etanercept has been used with some nonspecific benefit [33], while treatment with IL-1 blockers prevents disease relapse if administered for at least two weeks at the onset of fever [34].

## 5. NLRP-Related Diseases 

The cryopyrin-associated periodic syndromes (CAPS) encompass three diseases which represent a phenotypic spectrum, with mutations in the same *NLRP3* gene encoding cryopyrin, the master controller of caspase activation following numerous triggers, including invading pathogens and genotoxic stress. There is no apparent selective advantage demonstrated for CAPS.  Mutated cryopyrins can be found in at least 60% of patients, leading to the constitutive activation of the inflammasome and subsequent dysregulated IL-1 overproduction: excessive IL-1 signaling appears to be a constant feature in the background of CAPS, driven by gain-of-function *NLRP3 *mutations [35]. Familial cold autoinflammatory syndrome, Muckle-Wells syndrome, and neonatal onset multisystem inflammatory disorder (NOMID) have a significant symptom overlap, including fatigue, fever, and inflammation of the skin, eyes, bones, joints, and meninges, which might even reduce life expectancy [36]. Although rare, NOMID has been reported across the world and is the most severe expression of CAPS, with typical hypertrophic arthropathy involving both epiphyses of long bones and kneecaps, aseptic chronic meningitis, elevated intracranial pressure, deafness, and growth retardation [37]. General details of NLRP-related diseases, including the recently identified *NLRP12*-associated autoinflammatory disorder, are summarized in Table 4. Treatment had been quite disappointing until the impressive clinical results obtained with IL-1 antagonists, linking all CAPS specifically to IL-1 secretion and instituting IL-1 blockage as the gold standard treatment [38]. Anakinra, the recombinant IL-1 receptor antagonist, was the first biologic designed for the selective blockage of IL-1 [39], while canakinumab, a fully human monoclonal anti-IL-1beta antibody, has recently been registered for patients with CAPS, though the optimal administration schedule in terms of dosage and frequency of injections is still undetermined [40].

## 6. Skin Autoinflammatory Disorders

Different AIDs can be encountered in a dermatological setting, and several syndromes display a combination of recurrent early-onset fevers, multisystem inflammation, and cutaneous lesions, for which a skin biopsy might provide diagnostic insight [41].  Table 5 lists these conditions. Among pyogenic disorders, PAPA syndrome is defined by the triad of “pyogenic arthritis, pyoderma gangrenosum, and cystic acne”, arising from mutations in the *PSTPIP1* gene encoding a protein called “CD2 binding protein 1” and leading to disrupted interaction with pyrin and the inflammasome [42]: the first description of PAPA syndrome dates back to the 1997 report of an extended family with 10 affected members in three generations, although no geographical restriction was noted in the following reports. Potential treatment methods for patients with PAPA syndrome are corticosteroids, IL-1 antagonists, and tumor necrosis factor blocking agents, with varying results in terms of clinical effectiveness [43].

The deficiency of the interleukin-1 receptor antagonist, caused by loss-of-function mutations in the *IL1RN *gene, leads to unopposed proinflammatory IL-1 signaling and is revealed by pustular skin eruption and multifocal osteomyelitis, which occur in the neonatal period and respond excellently to anakinra. The first report describing this condition concerned 9 cases from the Netherlands, Canada, Lebanon, and Puerto Rico [44].

In 1985, Blau described the presence of a peculiar phenotype in 11 family members spanning four generations, characterized by asymptomatic ichthyosiform rash, deforming polyarthritides with boggy synovial effusions or cysts, and severe recurrent panuveitis: the disease was then called Blau syndrome and related to gain-of-function mutations in the *NOD2* gene, inherited in an autosomal dominant manner [45]. Systemic corticosteroids are the mainstay of therapy, but steroid-sparing agents such as methotrexate, tumor necrosis factor-inhibitors, or IL-1 antagonists have frequently been used [46].

Despite demonstration of the great efficacy of IL-1 blocking therapy in many AIDs, revealing innate immune system workings and the key role of IL-1 in autoinflammatory attacks, new AIDs have recently been recognized as unresponsive to IL-1 antagonists, including the proteasome-related “chronic atypical neutrophilic dermatitis with lipodystrophy and elevated temperature” syndrome, or CANDLE syndrome, which was initially described in different areas of Japan and named Nakajo-Nishimura syndrome [47]. The disease starts in the first year of life**  **with a skin eruption evolving to lipomuscular atrophy in the upper body, associated with periodic high fevers and has been recognized in different countries around the world, not only in the area of Japan. Joint contractures, muscle atrophy, and panniculitis-induced lipodystrophy have also been listed as crucial signs, and the disease is now included in the group of proteasome disability syndromes, a new category of AID caused by mutations in the *PSMB8* gene, encoding the *β*5i subunit of the immunoproteasome, the protease complex specialized for the degradation of polyubiquitinated proteins [48]. Interferon-gamma and interleukin-6 are the basic mediators of systemic inflammation observed in these patients, urging the continuation of research to characterize alternative subverted innate immune pathways in clinical medicine.

The up-to-date enhancement of genetic tools has led to the recognition of an increasing number of patients with AIDs and to a better description of multiple disease phenotypes. Genes involved in the regulation of the inflammatory response may also participate to the pathogenesis of different rare diseases with no precise etiology, which cannot be defined as AIDs, characterized by relapsing attacks: that is, intermittent hydrarthrosis, characterized by periodic episodes of painless joint swelling, unexpectedly associated with high frequency of heterozygous mutations in the *MEFV* gene [49], systemic capillary leak syndrome, in which spontaneous capillary leakage and shift of plasma from the intravascular to the extravascular space occur [50], and palindromic rheumatism, with idiopathic sudden-onset episodes of recurrent arthritis [51].

**Figure 1:**
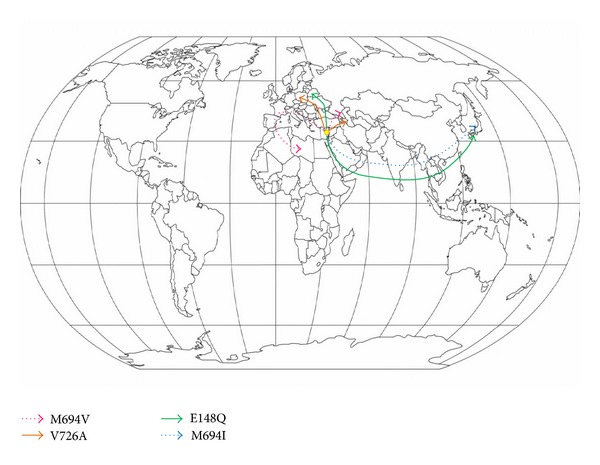
The migration of M694V, V726A, E148Q, and M694I *MEFV* mutations from Eastern Mediterranean areas may ideally explain some ethnically restricted prevalence data for familial Mediterranean fever.

Recently, a European AID registry, the “Eurofever registry”, has shown that a considerable number of patients with AIDs are of European ancestry [52], and many mutation profiles show differences when analyzed for distinct populations; that is, the *MEFV *E148Q mutation is frequently found in Europe, while the double heterozygosis E148Q/M694I is mainly observed in Japan [53]. FMF can provide an example of historical positive selection pressure, favouring heterozygosity of *MEFV *mutations: their extensive spread in an ideal “odyssey” through Arab conquests, the dispersal of the Armenian nation, the Jewish Diaspora, and the East-to-West European migration of recent years might reflect a better response to intracellular pathogens or a potential protection against still unknown diseases (Figure 1). A number of questions remain regarding the biologic basis of the heterozygote advantage for recessively inherited AID-related genes or the natural selection of mutated genes related to dominantly-inherited AID. Nevertheless, our final impression is that AIDs can be recognized across multiple ethnicities without any geographical restriction, and therefore, clinicians' awareness of AIDs must be reinforced across various medical specialties. Further research is ongoing with the aim of strengthening our comprehension of the genetics behind these heterogeneous conditions, personalizing therapies with anti-inflammatory cytokines or at least mitigating the intensity of the inflammatory cascade, and improving the destiny of children and adults with AIDs.

## 7. Conclusive Remarks

The study of AIDs, characterized by lifelong recurrent and seemingly unprovoked episodes of inflammation and fever, has revealed that specific conditions like FMF, MKD, TRAPS, NLRP-related diseases, and skin autoinflammatory disorders have distinct features and specific therapeutic options, emphasizing the need for a precise diagnostic identification in each case. Obtaining a family history is an essential part of the evaluation protocol of these patients, and priority is to examine proband's clinical and laboratory data both in the acute inflammatory phase and in the intercritical phase, in order to confirm the presence of a subclinical inflammation between attacks and exclude a host of chronic diseases of infectious, autoimmune, and even neoplastic nature. A substantial contribution to the diagnosis derives from both the consideration of ethnicity and genotype analysis related to *MEFV, MVK, TNFRSF1A, NLRP3, NLRP12, PSTPIP1, IL1RN, CARD15/NOD2, *and *PSMB8*, which are the genes responsible for the syndromes collectively termed AIDs. However, the diagnostic path for many of these patients remains often long and requires high-level expertise: the creation of international registries is highly needed to standardize guidelines about the role of genetics in the diagnosis and expedite the definition of the best treatment protocols.

## Figures and Tables

**Table 1:** The clinical features of familial Mediterranean fever.

Gene	*MEFV* (16p13.3)
Inheritance	Autosomal recessive (an autosomal dominant pattern has been reported)
Protein encoded	Pyrin
OMIM	249100 (134610 for the autosomal dominant variant)
Onset	Childhood or adolescence
Fever	Over 39°C Preceded by chills in 20% of cases Lasting few hours-3 days
Major clinical signs	Recurrent peritonitis, pleurisy, and pericarditis and/or synovitis in large joints
Rash features	Erysipelas-like erythema In the lower extremities
Gonadal involvement	Orchitis
Rare signs	Aseptic meningitis, cryptogenic cirrhosis, and Th1-polarized vasculitides
Major complication	Amyloidosis of AA type (the risk differs according to ethnic group)
Major “*in vivo*” diagnostic test	Favorable response to daily colchicine administration
Treatment	Colchicine, interleukin-1 antagonists

**Table 2:** The clinical features of mevalonate kinase deficiency.

Gene	*MVK* (12q24)
Inheritance	Autosomal recessive
Protein encoded	Mevalonate kinase
OMIM	260920 (610377 for mevalonic aciduria)
Onset	First infancy
Fever	Over 40°C Preceded by chills and malaise Lasting 4–7 days
Gastrointestinal signs	Severe abdominal pain, vomiting, and diarrhoea
Rash features	Macular, papular, morbilliform, nodular, and urticarial, but also resembling Henoch-Schönlein purpura, erythema nodosum, or erythema elevatum diutinum
Mucosal signs	Oral and/or vaginal aphthous ulcers in 50% of patients
Articular signs	Symmetrical arthralgias, nonerosive arthritides in large joints
Lymph node involvement	Cervical or diffuse Bilateral Painful
Visceral involvement	Hepatosplenomegaly
Neurologic involvement	Nonspecific headache
Serum marker in the interfebrile phase	IgD above 100 IU/mL (in at least 80% of patients)
Urinary marker during febrile attacks	Increased excretion of mevalonic acid
Treatment	Anti-inflammatory drugs, corticosteroids, and on-demand interleukin-1 antagonists

**Table 3:** The clinical features of tumor necrosis factor receptor-associated periodic fever syndrome.

Gene	*TNFRSF1A* (12p13)
Inheritance	Autosomal dominant
Protein encoded	p55 receptor of tumor necrosis factor
OMIM	142680
Onset	Infancy or adolescence
Fever	Over 39°C Recurring at least 2–6 times each year Absent in a subset of patients
Duration of fever episodes	Prolonged (from 1 week to 4 weeks)
Gastrointestinal signs	Severe abdominal pain, vomiting, and constipation
Rash features	Erythematous and migratory (with centrifugal trend) Cellulitis-like plaques and serpiginous patches Painful
Muscular signs	Focal myositis, monocytic fasciitis Cramping muscular pain Migratory (with centrifugal trend)
Joint involvement	Arthralgia, tenosynovitis
Ocular signs	Periorbital edema, aseptic painful conjunctivitis, and uveitis
Serosal signs	Pleuritis, pericarditis
Major complication	Amyloidosis of AA type
Serum marker in the interfebrile phase	Soluble TNF receptor (TNFRSF1A) lower than 1 ng/mL
Treatment	Interleukin-1 antagonists

**Table 4:** The clinical features of NLRP-related diseases.

	Familial cold autoinflammatory syndrome	Muckle-Wells syndrome	Neonatal onset multisystem inflammatory disorder	NLRP12-associated autoinflammatory disorder (or familial cold autoinflammatory syndrome 2)
Gene	*NLRP3 *(1q44)			*NLRP12 *(19q13.42)
Inheritance	Autosomal dominant			
Protein encoded	Cryopyrin			Monarch 1
OMIM	120100	191900	607115	609648
Onset age	Infancy	Infancy-adolescence	Prenatal period	Infancy
Duration of clinical signs	Less than 24 hours	Subcontinuous	Continuous	Periodic
Fever	Short duration	Recurrent	Recurrent	Periodic
Rash features	Cold-induced, urticaria-like	Cold-induced, urticaria-like, and evanescent	Migratory, polymorphous, and urticaria-like	Cold-induced, urticaria-like
Ocular signs	Conjunctivitis	Conjunctivitis	Chronic papilledema, optic nerve atrophy, and visual loss	—
Muscular-skeletal symptoms	Arthralgia, transient joint stiffness	Lifelong arthralgias, nonerosive arthritides, and chronic fatigue	Deforming osteoarthropathy of large joints (with abnormal ossification of patellae)	Arthralgia
Neurologic signs	—	Risk of sensorineural deafness	Chronic aseptic meningitis, sensorineural deafness	Headache, sensorineural deafness
Major complication	Amyloidosis of AA type			
Treatment	Interleukin-1 antagonists (canakinumab)			Interleukin-1 antagonists

**Table 5:** Dermal pathology of skin autoinflammatory disorders.

	PAPA syndrome	Deficiency of interleukin-1 receptor antagonist	Blau syndrome	CANDLE syndrome (Nakajo-Nishimura syndrome)
Gene	*PSTPIP1 *(15q24-q25.1)	*IL1RN *(2q)	*CARD15/NOD2 *(16q2)	*PSMB8 *(6p21.3)
Inheritance	Autosomal dominant	Autosomal recessive	Autosomal dominant	Autosomal recessive
Protein encoded	CD_2_ antigen-binding protein 1	Interleukin-1 receptor antagonist	NOD2	*β*5i subunit of the immunoproteasome
OMIM	604416	612852	186580	256040
Onset	Infancy	Neonatal period	Before 3-4 years	Infancy
Fever	—	Absent	Inconstant	Recurrent
Rash features	Pyoderma gangrenosum, cystic acne	Pustular with ichthyosis-like changes	Brown-coloured and flaky, multiple subcutaneous granulomatous nodules	Cold-induced pernio-like or purpuric/vasculitic lesions, progressive lipodystrophic changes in the upper body
Articular signs	Sterile pyogenic oligoarthritis	Sterile multifocal osteomyelitis	Recurrent granulomatous symmetric polyarthritis with progressive trend, tenosynovial cysts, and “boutonnière” finger deformities	Joint contractures, clubbed fingers and toes
Ocular signs	—	—	Recurrent granulomatous panuveitis, chorioretinitis	—
Neurologic signs	—	—	—	Basal ganglia calcification
Treatment	Corticosteroids, tumor necrosis factor-inhibitors, and interleukin-1 antagonists	Interleukin-1 antagonists	Corticosteroids, immunosuppressive agents, tumor necrosis factor-inhibitors, and interleukin-1 antagonists	Corticosteroids, dapson, interferon-gamma modulators, and interleukin-6 antagonists

## References

[1] Hashkes PJ, Toker O (2012). Autoinflammatory syndromes. *Pediatric Clinics of North America*.

[2] Lamkanfi M, Dixit VM (2012). Inflammasomes and their roles in health and disease. *Annual Review of Cell and Developmental Biology*.

[3] Rigante D (2012). The fresco of autoinflammatory diseases from the pediatric perspective. *Autoimmunity Reviews*.

[4] Ben-Chetrit E, Touitou I (2009). Familial Mediterranean fever in the world. *Arthritis Care and Research*.

[5] Guz G, Kanbay M, Ozturk MA (2009). Current perspectives on familial Mediterranean fever. *Current Opinion in Infectious Diseases*.

[6] Ben-Chetrit E, Levy M (1998). Familial Mediterranean fever. *The Lancet*.

[7] Rigante D, La Torraca I, Ansuini V, Compagnone A, Sallì A, Stabile A (2006). The multi-face expression of familial Mediterranean fever in the child. *European Review for Medical and Pharmacological Sciences*.

[8] Cattan D (2003). Familial Mediterranean fever: is low mortality from tuberculosis a specific advantage for *MEFV* mutations carriers? Mortality from tuberculosis among Muslims, Jewish, French, Italian and Maltese patients in Tunis (Tunisia) in the first half of the 20th century. *Clinical and Experimental Rheumatology*.

[9] Papadopoulos VP, Giaglis S, Mitroulis I, Ritis K (2008). The population genetics of familial Mediterranean fever: a meta-analysis study. *Annals of Human Genetics*.

[10] Jalkh N, Génin E, Chouery E (2008). Familial Mediterranean fever in Lebanon: founder effects for different *MEFV* mutations. *Annals of Human Genetics*.

[11] Tsuchiya-Suzuki A, Yazaki M, Nakamura A (2009). Clinical and genetic features of familial Mediterranean fever in Japan. *Journal of Rheumatology*.

[12] Ebrahimi-Fakhari D, Schِnland SO, Hegenbart U (2013). Familial Mediterranean fever in Germany: clinical presentation and amyloidosis risk. *Scandinavian Journal of Rheumatology*.

[13] Touitou I, Sarkisian T, Medlej-Hashim M (2007). Country as the primary risk factor for renal amyloidosis in familial Mediterranean fever. *Arthritis and Rheumatism*.

[14] Sohar E, Gafni J, Pras M, Heller H (1967). Familial Mediterranean fever. A survey of 470 cases and review of the literature. *The American Journal of Medicine*.

[15] Samuels J, Aksentijevich I, Torosyan Y (1998). Familial Mediterranean fever at the millennium clinical spectrum, ancient mutations, and a survey of 100 American referrals to the national institutes of health. *Medicine*.

[16] Livneh A, Langevitz P, Zemer D (1997). Criteria for the diagnosis of familial Mediterranean fever. *Arthritis and Rheumatism*.

[17] Soriano A, Manna R (2012). Familial Mediterranean fever: new phenotypes. *Autoimmunity Reviews*.

[18] Rigante D, La Torraca I, Avallone L, Pugliese AL, Gaspari S, Stabile A (2006). The pharmacologic basis of treatment with colchicine in children with familial Mediterranean fever. *European Review for Medical and Pharmacological Sciences*.

[19] Roldan R, Ruiz AM, Miranda MD, Collantes E (2008). Anakinra: new therapeutic approach in children with familial Mediterranean fever resistant to colchicine. *Joint Bone Spine*.

[20] van der Meer JWM, Vossen JM, Radl J (1984). Hyperimmunoglobulinemia D and periodic fever: a new syndrome. *The Lancet*.

[21] Lachmann HJ (2011). Clinical immunology review series: an approach to the patient with a periodic fever syndrome. *Clinical and Experimental Immunology*.

[22] Vuch J, Marcuzzi A, Bianco AM, Tommasini A, Zanin V, Crovella S (2013). Evolutionary hypothesis of the mevalonate kinase deficiency. *Medical Hypotheses*.

[23] Drenth JPH, Haagsma CJ, van der Meer JWM (1994). Hyperimmunoglobulinemia D and periodic fever syndrome. The clinical spectrum in a series of 50 patients. *Medicine*.

[24] Ammouri W, Cuisset L, Rouaghe S (2007). Diagnostic value of serum immunoglobulinaemia D level in patients with a clinical suspicion of hyper IgD syndrome. *Rheumatology*.

[25] Rigante D, Ansuini V, Bertoni B (2006). Treatment with anakinra in the hyperimmunoglobulinemia D/periodic fever syndrome. *Rheumatology International*.

[26] Bodar EJ, Kuijk LM, Drenth JPH, van der Meer JWM, Simon A, Frenkel J (2011). On-demand anakinra treatment is effective in mevalonate kinase deficiency. *Annals of the Rheumatic Diseases*.

[27] Drenth JPH, van Deuren M, van der Ven-Jongekrijg J, Schalkwijk CG, van der Meer JWM (1995). Cytokine activation during attacks of the hyperimmunoglobulinemia D and periodic fever syndrome. *Blood*.

[28] Aksentijevich I, Galon J, Soares M (2001). The tumor-necrosis-factor receptor-associated periodic syndrome: new mutations in TNFRSF1A, ancestral origins, genotype-phenotype studies, and evidence for further genetic heterogeneity of periodic fevers. *The American Journal of Human Genetics*.

[29] Nedjai B, Hitman GA, Church LD (2011). Differential cytokine secretion results from p65 and c-Rel NF-*κ*B subunit signaling in peripheral blood mononuclear cells of TNF receptor-associated periodic syndrome patients. *Cellular Immunology*.

[30] Ryan JG, Goldbach-Mansky R (2008). The spectrum of autoinflammatory diseases: recent bench to bedside observations. *Current Opinion in Rheumatology*.

[31] Stojanov S, McDermott MF (2005). The tumour necrosis factor receptor-associated periodic syndrome: current concepts. *Expert Reviews in Molecular Medicine*.

[32] Dinc A, Erdem H, Rowczenio D (2005). Autosomal dominant periodic fever with AA amyloidosis: tumor necrosis factor receptor-associated periodic syndrome (TRAPS) in a Turkish family. *Journal of Nephrology*.

[33] Cantarini L, Rigante D, Lucherini OM (2010). Role of etanercept in the treatment of tumor necrosis factor receptor-associated periodic syndrome: personal experience and review of the literature. *International Journal of Immunopathology and Pharmacology*.

[34] Gattorno M, Pelagatti MA, Meini A (2008). Persistent efficacy of anakinra in patients with tumor necrosis factor receptor-associated periodic syndrome. *Arthritis and Rheumatism*.

[35] Cantarini L, Lucherini OM, Frediani B (2011). Bridging the gap between the clinician and the patient with cryopyrin-associated periodic syndromes. *International Journal of Immunopathology and Pharmacology*.

[36] Maksimovic L, Stirnemann J, Caux F (2008). New CIAS1 mutation and anakinra efficacy in overlapping of Muckle-Wells and familial cold autoinflammatory syndromes. *Rheumatology*.

[37] Aksentijevich I, Putnam CD, Remmers EF (2007). The clinical continuum of cryopyrinopathies: novel CIAS1 mutations in North American patients and a new cryopyrin model. *Arthritis and Rheumatism*.

[38] Yu JR, Leslie KS (2011). Cryopyrin-associated periodic syndrome: an update on diagnosis and treatment response. *Current Allergy and Asthma Reports*.

[39] Rigante D, Leone A, Marrocco R, Laino ME, Stabile A (2011). Long-term response after 6-year treatment with anakinra and onset of focal bone erosion in neonatal-onset multisystem inflammatory disease (NOMID/CINCA). *Rheumatology International*.

[40] Lachmann HJ, Kone-Paut I, Kuemmerle-Deschner JB (2009). Use of canakinumab in the cryopyrin-associated periodic syndrome. *The New England Journal of Medicine*.

[41] Rigante D, Cantarini L (2011). Monogenic autoinflammatory syndromes at a dermatological level. *Archives of Dermatological Research*.

[42] Tallon B, Corkill M (2006). Peculiarities of PAPA syndrome. *Rheumatology*.

[43] Demidowich AP, Freeman AF, Kuhns DB (2012). Brief report: genotype, phenotype, and clinical course in five patients with PAPA syndrome (pyogenic sterile arthritis, pyoderma gangrenosum, and acne). *Arthritis and Rheumatism*.

[44] Aksentijevich I, Masters SL, Ferguson PJ (2009). An autoinflammatory disease with deficiency of the interleukin-1-receptor antagonist. *The New England Journal of Medicine*.

[45] Blau EB (1985). Familial granulomatous arthritis, iritis, and rash. *Journal of Pediatrics*.

[46] Sfriso P, Caso F, Tognon S, Galozzi P, Gava A, Punzi L (2012). Blau syndrome, clinical and genetic aspects. *Autoimmunity Reviews*.

[47] Kanazawa N (2012). Nakajo-Nishimura syndrome: an autoinflammatory disorder showing pernio-like rashes and progressive partial lipodystrophy. *Allergology International*.

[48] Torrelo A, Patel S, Colmenero I (2010). Chronic atypical neutrophilic dermatosis with lipodystrophy and elevated temperature (CANDLE) syndrome. *Journal of the American Academy of Dermatology*.

[49] Cañete JD,  Aróstegui JI, Queiró R (2006). Association of intermittent hydrarthrosis with *MEFV* gene mutations. *Arthritis and Rheumatism*.

[50] Piastra M, Pietrini D, Conti G, de Rosa G, Rigante D (2012). Sudden shock from capillary leak. *The Lancet*.

[51] Cañete JD, Arostegui JI, Queiró R (2007). An unexpectedly high frequency of *MEFV* mutations in patients with anti-citrullinated protein antibody-negative palindromic rheumatism. *Arthritis and Rheumatism*.

[52] Haar NT, Lachmann H, Ozen S (2013). Treatment of autoinflammatory diseases: results from the eurofever registry and a literature review. *Annals of the Rheumatic Diseases*.

[53] Migita K, Uehara R, Nakamura Y (2012). Familial Mediterranean fever in Japan. *Medicine*.

